# Occurrence and sources of hormones in water resources—environmental and health impact

**DOI:** 10.1007/s11356-024-33713-z

**Published:** 2024-05-21

**Authors:** Martyna Grzegorzek, Katarzyna Wartalska, Robert Kowalik

**Affiliations:** 1 Faculty of Environmental Engineering, Wroclaw University of Science and Technology, Wybrzeze Stanisława Wyspianskiego 27, 50-370 Wroclaw, Poland; 2 Faculty of Environmental Engineering, Geodesy and Renewable Energy, Kielce University of Technology, Al. Tysiąclecia Państwa Polskiego 7, 25-314 Kielce, Poland

**Keywords:** Hormones, Sources, Hormone classification, Health, Environment, Environmental impact

## Abstract

Within recent years, hormones have become emergent contaminants in the water environment. They easily accumulate in living organisms which in effect leads to numerous health problems (endocrine-disrupting mechanism is one of the most known toxic effects). Microbial resistance to antibiotics also became one of the emergent issues related to hormone presence. It was shown that the most common in the environment occur estrogens (E1, E2, E3, and EE2). It has been proven that large amounts of hormones are released from aquaculture as well as from wastewater treatment plants (due to the relatively low separation efficiency of conventional wastewater treatment processes). Within the article’s scope, the literature review was performed. The analysis was regarding the characterization of the hormone substances present in the environment, their influence on living organisms and the environment, as well as its potential sources classification.

## Introduction

Hormones, as key regulators of biological processes, play an extremely important role in the life of aquatic organisms, as well as in the ecosystems that coexist with them (Tao and Cheng 2023). However, the emergence and presence of hormones in aquatic resources is becoming increasingly visible, and their sources and impacts on the environment and public health are becoming the subject of in-depth analysis and interest (Ojoghoro et al. 2021).

In recent years, there has been a growing awareness of the impact of endocrine substances, such as hormones, on aquatic ecosystems. The sources of these substances are diverse and include both natural biological processes and human activities (Thacharodi et al. 2023). Pharmaceutical products, contraceptives, hormones used in animal husbandry, and industrial by-products are just a few of the many sources that can introduce these substances into the aquatic environment (Raj et al. 2023).

With social, economic, and industrial development, more and more new anthropogenic pollutants appear in the environment. These pollutants enter drinking water resources (surface and groundwater), which raises questions related to not only ecology, but also human health and economic impacts (Benner et al. 2013; Schröder et al. 2016). According to the literature, between 30,000 and 70,000 chemicals, out of over 100,000 registered in the European Union, are found in everyday human life. These include compounds such as fragrances, pharmaceuticals, petrochemicals, hormones, pesticides, personal care products, additives, and disinfection products (Besha et al. 2017; Bozyigit et al. 2022). The presence of these contaminants in the environment is connected mainly to domestic sewage disposal, agricultural runoffs, industrial, and wastewater treatment plant (WWTP) effluents (Rezka et al. 2015; Lei et al. 2020; Sacdal et al. 2020; Ribeiro et al. 2021). If the drugs are not degraded or eliminated during sewage treatment, in soil or in other environmental compartments, they will reach surface water and groundwater, and, potentially, drinking water (Hartmann et al. 2014). Hormones are compound which are responsible for contamination water resources worldwide. Among other, analyses performed by the USA, Canada, Brazil, and European Union countries indicate the presence of over 80 pharmaceutical substances in water (Wontorska 2018; Almazrouei et al. 2023).

The controversial nature of the presence of certain groups of pharmaceuticals depends not so much on the level of concentrations in which they are present in water bodies, but mainly on their impact on aquatic organisms and humans. Hence, the presence of hormonal substances has been much more frequently raised than other drug residues in recent decades (Rezka et al. 2015). The presence of natural and synthetic hormones in the aquatic environment is therefore becoming a growing concern, with potential negative impacts not only for aquatic organisms, but also for people and animals. Hormones may affect the growth, development, and reproduction of aquatic organisms (Matthiesen 1998; Ribeiro et al. 2021). Living organisms exposed to constant influence of hormones may experience problems related to endocrine disorders.

Steroid hormones, such as estrogens, androgens, progestogens, glucocorticoids, and mineralocorticoids, are frequently found in the environment (Chang et al. 2009; Ribeiro et al. 2021). A significant increase in the use of pharmaceuticals in recent years translates into an increase in the problem of labeling them and determining their concentration levels in the natural environment. Importantly, we are now seeing the appearance of new compounds, previously not detected in water bodies, as well as an increase in the concentration of hormones previously present in much lower concentrations. The level of hormones detected in surface waters is typically in the concentration range of ng/dm^3^ and µg/dm^3^, which allows these compounds to be classified as nano- and microcontaminants (Molnár et al. 2022).

In the light of the United Nations Sustainable Development Goals (SDGs) (Huck 2023), in particular SDG6 *Clean Water and Sanitation* as well as SDG11 which is *Sustainable Cities and Communities*, it is necessary to implement actions to mitigate hormone pollution in water and protect both human health and the environment. To address these issues, there is a need for effective treatment technologies, infrastructure repair and maintenance, water conservation, and strict pollution control laws (Chowdhary et al. 2020).

There are numerous studies investigating the fate and transport of hormones in the environment, as well as the effectiveness of their removal in water and wastewater treatment processes. Its presence is leading to significant water quality deterioration what decreases the volume of available water resources resulting in enhancing the problem of water scarcity. The literature most often mentions wastewater treatment plants as the main source of pollution, while other sources of water pollution with pharmaceuticals are rarely discussed. There is still a lack of studies constituting reviews regarding characteristics of hormones (their structure, presence of particular groups in the aquatic environment, the acceptable concentrations), sources of hormones in the aquatic environment and the influence on health and environment. Although more research was undertaken on hormone occurrence in water, many of them are case studies and are focused on specific aspects or presented with a high degree of generality. The information is scattered throughout many publications. The aim of the article is a systematic and comprehensive review of the hormones occurring in the environment, its sources and impact on living organisms. The innovation of the review lies in the synthesis of all these aspects in one paper, while these topics usually state separate publications. The first part of the work summarized the types of these compounds present in water. The second part of the work presents the sources of water pollution with hormones. The third part describes the impact that hormones have on living organisms.

## Characteristics of hormones: structure, dominant hormones in the aquatic environment, and their acceptable concentrations

Hormones are chemical compounds produced by glands in animal and human bodies that regulate a variety of physiological functions by transmitting signals to various cells in the body. These chemical compounds are produced by various glands in the body, such as endocrine glands (e.g., pituitary, thyroid, adrenal glands), as well as by other tissues such as intestinal cells or sex glands (Adeel et al. 2017; Cheng et al. 2020; Guerrero-Gualan et al. 2023).

Hormones regulate many processes in the body, such as growth and development, metabolism, sexual function, water and electrolyte balance, stress responses, body temperature control, and many other physiological functions. They affect various tissues and organs, controlling their activity and function through interactions with receptors on the cell surface. Estrogens are a group of steroid hormones that play a key role in regulating the development and function of female sexual characteristics and other physiological processes.

The content of estrogens in wastewater depends on the size of the human population living in a given area, as well as the number of women in the reproductive period, menopausal period, and pregnant women. The body of a pregnant woman produces up to 120 times more of the hormone 17β-estradiol than the body of a menopausal woman. Table 1 shows the average daily production of estrogens by humans (Farré et al. 2008; Vulliet and Cren-Olivé 2011).

**Table 1:** The amount of average steroid estrogen (E1, 17β-E2, and E3) excretion by human bodies (per person) in µg/day (Adeel et al. 2017; Grdulska and Kowalik 2020)

	Estrone (E1)	17β-estradiol (17β-E2)	Estriol (E3)	Reference
Average per person	19.00	7.70	8100	(Laurenson et al. 2014)
Women				
On average	7.00	2.40	4.40	(Andaluri et al. 2012)
Pregnant	787	277	9850	(Kostich et al. 2013)
Menstruating	9.32	6.14	17.40	(Kostich et al. 2013)
	3.50	8.00	4.80	(Kostich et al. 2013)
Menopausal	2.30	4.00	1.00	(Hamid and Eskicioglu 2012)
Menopausal, no HRT*	2.93	1.49	3.90	(Kostich et al. 2013)
Menopausal, with HRT*	31.50	59.20	90.70	(Kostich et al. 2013)
Males				
On average	1.60	3.90	1.50	(Hamid and Eskicioglu 2012)
Adult male	3.50	1.83	3.21	(Kostich et al. 2013)
Children				
Female	0.60	2.50	0.918	(Kostich et al. 2013)
Male	0.63	0.54		(Kostich et al. 2013)

*HRT hormone replacement therapy

The presence of these steroidal estrogens has been globally confirmed by researchers, not only in groundwater, but also in freshwater. It has been reported that the livestock industry discharges about 58% of estradiol (E2), which is excreted in urine; on the other hand, the percentage of 17α-ethinylestradiol (EE2) and estriol (E3) is about 96% and 69%, respectively (Pauwels et al. 2008b; Dai et al. 2022).

Concentrations of E1 in surface waters tend to be higher than other natural estrogens, including E2 and E3, in many countries around the world. This may be due to the higher excretion rate of E1 from organisms and the transformation of E2 to E1. Although the synthetic estrogen, EE2, is mostly below the detection limit of surface water (Table 3), it has been found to contribute to most of the excreted (Laurenson et al. 2014). Given that EE2 is more persistent than E1, E2, and E3, greater attention to the detection and removal of EE2 from surface waters is necessary estrogenicity (Khanal et al. 2007; Xu et al. 2018).

Four of the most common natural and synthetic estrogens (E1, E2, E3, and EE2), their physical properties and structure are shown in Table 2.

**Table 2:**
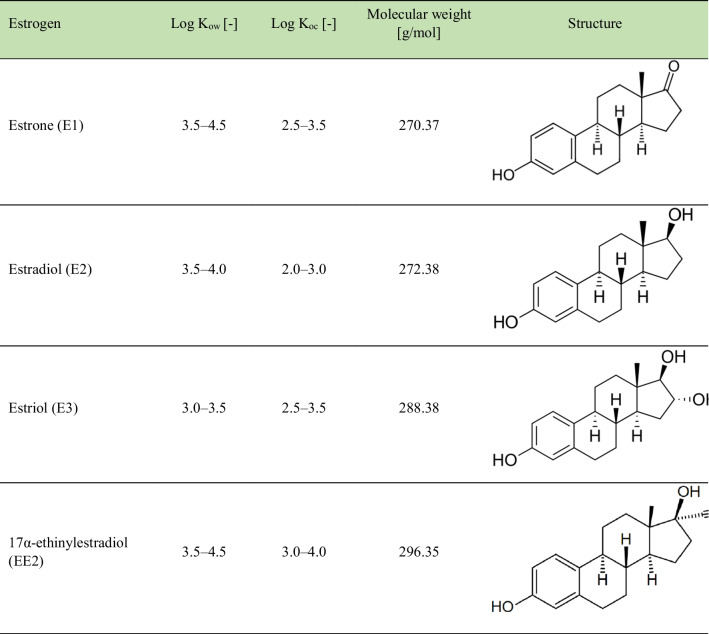
Physical properties of selected estrogens (E1, E2, E3, EE2) (Pauwels et al. 2008b; Xu et al. 2018).

The Log K_ow_ (logarithm of the octanol–water partition coefficient) is an indicator that determines the distribution of a substance between the organic (octanol) and aqueous phases. The Log K_ow_ reflects the tendency of a substance to dissolve in the organic phase compared to the aqueous phase. Log K_ow_ values for estrogens suggest moderately fairly good solubility in the organic phase compared to the aqueous phase, which may influence their potential distribution and behavior in the environment (Dias et al. 2021; Qiao et al. 2023).

The Log K_oc_ index (log organic sorption coefficient) refers to the ability of chemicals to sorb to organic matter in the soil. Log K_oc_ values can be used to estimate the potential mobility of a substance in the soil and possible entry into groundwater. However, it should be noted that the exact Log K_oc_ values can vary depending on many factors, including environmental conditions and soil composition (Ukalska-Jaruga et al. 2023).

In humans and animals, the estrogens E1, E2, and E3, which are female hormones, are derived from primary substrates such as cholesterol and pregnenolone, forming a key fragment of the common cyclopentane-perhydrophenanthrene ring (Dembitsky 2023). They function as important regulators of reproductive tissue health, breast, skin, and brain function. The synthetic steroid EE2 is commonly used as an oral contraceptive. Excess estrogens can lead to abnormal binding to estrogen receptors, interfering with normal biological processes. Estrogens E1, E2, E3, and EE2 share a common tetracyclic structure, consisting of one phenolic ring, two cyclohexane rings, and one cyclopentane ring. The differences between them are due to the fourth ring at positions C16 and C17. E1 is characterized by the presence of a carbonyl group at C17, E2 has a hydroxyl group at C17, E3 contains two alcohol groups at C16 and C17, and EE2 has a hydroxyl group and an ethinyl group at C17. The C17 hydroxyl group on E2 can be directed downwards on the molecular plane, resulting in an isomeric form bound to C17, or be directed upwards, forming the compound E2 (Fernandez et al. 2007; Chiang et al. 2020; Datel and Hrabankova 2020). These subtle structural differences affect their interactions with estrogen receptors and their biological functions.

It is necessary to establish national and international standards to regulate the environmental impact of steroid hormones from wastewater treatment plants. There are few international and national standards in the literature for hormones in wastewater or even drinking water. In 2008, the United States Geological Survey (USGS) began work to establish some standards across the country. They tested water in nine states and found 85 artificial chemicals, including some drugs. Hormones in water supplies are usually in insufficient concentrations, such as parts per billion (ppb) or parts per trillion (ppt), while their sources are homes, agriculture, industry, livestock, and landfills (World Health Organization 2011; Hrkal et al. 2018).

The US Environmental Protection Agency and the World Health Organization (WHO) only provide threshold values for concentrations of endocrine compounds in wastewater as a threshold value of less than 1 ng/dm^3^ (Commission 2019). According to the World Health Organization (WHO), there are currently no specific, strict limits for estrogen content in drinking water. The WHO does not set specific limits for individual estrogenic substances such as estradiol, estrone, or ethinylestradiol as a standard for all countries. For chemicals such as estrogens that may be present in drinking water or the environment, the WHO recommends ongoing scientific research to assess public health risks and develop water quality guidelines (Organization 2012; Godoy et al. 2015; EC 2019).

The UK Environment Agency (UKEA) has also developed thresholds for steroids, particularly for estrogens, by surveying 90 different surface waters. In particular, to protect marine life, they set thresholds for estrogens, such as 0.1 ng/dm^3^ for EE2, 1 ng/dm^3^ for 17β-E2, and 3 ng/dm^3^ for E1 (Almazrouei et al. 2023). This also helped in the development of total maximum daily loads (TMDLs). Operating endocrine-disrupting compounds (EDCs) individually or independently is very difficult. In most cases, they are in group form for effluent removal (Luo et al. 2014; Almazrouei et al. 2023).

Table 3 shows estrogen concentrations in effluents flowing into and out of sewage treatment plants, as well as concentrations in Polish rivers.

**Table 3:** Estrogen concentrations in influents and effluents from WWTPs (Anna Szymonik 2013; Włodarczyk-Makuła 2016)

Estrogen	Wastewater treatment plant influents (ng/dm^3^)	Wastewater treatment plant effluents (ng/dm^3^)	Detection limits (ng/dm^3^)	Hormone content in Polish rivers (ng/dm^3^)		
				Odra	Kanał Gliwicki	Wisła
Estrone (E1)	0.5–670.0	0.2–196.7	0.19	1.3	1.1	lod
Estradiol (E2)	0.6–188.7	0.5–64.0	0.39	lod	lod	1.3
Estriol (E3)	0.5–80.0	0.4–39.1	0.24	lod	lod	lod
17α-ethinylestradiol (EE2)	0.5–19.9	0.59–5.6	0.33	lod	lod	lod

*lod limit of detection

Sources of hormones in the aquatic environment

## Sources of hormones in the aquatic environment

The water around the world is exposed to contamination by steroid hormones. Although hormones are released into the environment naturally (when people or animals take medicines containing synthetic estrogens, their bodies process these substances and some of them are excreted from the body through urine or feces), significant amounts of these substances are also related to human activities. The sources of hormones in the aquatic environment include municipal, industrial, and hospital sewage, wastewater treatment plants (some of these substances are hardly removed in conventional sewage water treatment), as well as animal breeding, agriculture and fish farms (aquaculture), pharmaceutical or cosmetic industry, and production processes can release these substances into the environment through water, air, or soil pollution. Cemeteries and landfills are also mentioned (Jiang et al. 2013; Rezka et al. 2015; Schröder et al. 2016; Zhang et al. 2022).

It is estimated that the human population excretes 30,700 kg of natural and synthetic estrogens (data from 2017). However, this amount constitutes only 37% of the hormones excreted by livestock in the USA and the European Union—83,000 kg (Adeel et al. 2017; Dai et al. 2022). In the European Union, the total excretion of endogenous estrogens, androgens, and progestins amounted to 356,700 kg, while in the USA—332,400 kg (data from 2000) (Lange et al. 2002; Dai et al. 2022).

Hormones are largely released into the environment along with treated sewage discharged from wastewater treatment plants and from sewage from places where livestock is fed (Behera et al. 2011; Adeel et al. 2017). This is because hormones are naturally excreted by the endocrine glands of both humans and animals (Shore and Shemesh 2016). When comparing the average levels of steroid estrogen excretion by humans (Table 1), the levels of hormones excreted by pregnant women are much higher than the levels of estrogen excreted by menopausal women. It is noticeable that the rate of excretion by non-pregnant women, males, and young children are progressively and substantially lower.

In addition to natural hormones, there are also synthetic ones in the environment, produced by the pharmaceutical industry, but also originating from veterinary offices, medical universities, and care facilities. These include agents used in pharmacology as drugs, including diethylstilbestrol (DES)—an estrogen prescribed in the past to pregnant women, and ethinylestradiol (EE2)—a drug that prevents miscarriages and improves pregnancy outcomes. There is also a group of hormones used in birth control and hormone replacement therapy (HRT) (Reed and Fenton 2013). The use of steroid hormones in pharmaceuticals, personal care products, livestock, and husbandry significantly pollutes water resources. A relatively new source of hormones in the environment and sewage are detergents, shampoos, and lotions (Roudbari and Rezakazemi 2018).

In the stream of sewage originating from human settlements, sewage from hospitals should be distinguished. Hospitals use significant amounts of water daily, far exceeding typical water consumption standards per person and day. While on average one person uses 100–200 dm^3^ of water per day, in hospitals it is 400 to 1200 dm^3^ per day and per bed (Emmanuel et al. 2002). This results in a significant outflow of sewage containing microorganisms, heavy metals, toxic chemicals, and hormones. Hospitals produce wastewater that combines characteristics of domestic, industrial, and medical wastewater (Nagarnaik et al. 2010). Indeed, several studies revealed that steroidal estrogens, especially high levels of estriol, were detected in all hospital sewage samples (Avberšek et al. 2011).

After excretion, hormones are discharged into sewage systems, and due to leaks in these networks, hormones may be released into the ground along with sewage exfiltration (Bexfield et al. 2019). Despite bans, expired medicines, excess or no longer needed medicines, which may contain hormones, also often end up in the sewage system. In the absence of sewage systems, the source of potential hormone contamination are septic tanks, where sewage may seep into soil and groundwater (Dudziak 2004).

Wastewater treatment plants are one of the main sources of hormone contamination in surface waters (Desbrow et al. 1998; Chang et al. 2011; Sacdal et al. 2020). Estrogens, specifically estrone (E1), 17β-estradiol (E2), estriol (E3), and ethinylestradiol (EE2), have been detected in numerous studies of wastewater influents and effluents (Caldwell et al. 2010). This is due to the fact that sewage treatment plants are unable to completely remove hormones from sewage, which leads to these substances being discharged into the natural environment along with the WWTP effluent (Pal et al. 2010; Andaluri et al. 2012; Belhaj et al. 2015; Adeel et al. 2017). According to many studies, only 27% of micropollutants can be removed from most wastewater treatment plants to a degree below the detection limit, while the remaining micropollutants include some that cannot be removed at all (Bolong et al. 2009; Combalbert and Hernandez-Raquet 2010; Chi et al. 2013). Hormone removal efficiency ranges from 0 to 99%, depending on country, location, and concentration of these micropollutants (Wojnarowicz et al. 2014; Blair et al. 2015). The research presented in Manickum and John (2014) showed the presence of estrogens in the wastewater treatment plant effluent from 1 ng/dm^3^ (EE2) to 107 ng/dm^3^ (E2), with their removal efficiency amounting to 90 ± 3% and 78 ± 12%, respectively. The release of such hormone concentrations into the river resulted in a distinct increase in levels of these compounds (in the case of estradiol—28 ng/dm^3^ above and 66 ng/dm^3^ below the WWTP).

A summary of the research results of the individual types of hormone concentrations in the wastewater treatment plant effluent is presented in Yazdan’s work (Yazdan et al. 2022). The hormone concentrations both in WWTP inflow and outflow are influenced by the characteristics of the municipal sewage catchment area, commerce-industry-domestic sewage mix, and the wastewater treatment technology used (Johnson and Sumpter 2001; Mispagel et al. 2009). The effectiveness of the secondary and tertiary treatment in reducing the concentrations of endocrine-active compounds in municipal sewage has been confirmed (Bain et al. 2014).

The impact of combined sewer overflows (CSOs) on the release of hormones into the aquatic environment was also investigated. It turns out that they are a significant source of hormones and micropollutants (Chang et al. 2011; Nickel and Fuchs 2019). The work (Phillips et al. 2012) proved that discharges from CSOs constitute only 10% of the total annual water discharge (CSO and WWTP treatment (> 90%)). With low pollutant removal efficiency (< 90%), less than 10% of the annual load comes from CSO. Concentrations of estrogens, androgens, and WMPs may be 10 times higher in CSO discharges compared to treated wastewater discharges. The amount of flow, both through the storm overflow and through the treatment plant, is also important. In the case of CSO, as the flow increases, the concentration of pollutants decreases due to the greater degree of dilution of the sewage by precipitation. The opposite is the case with the inflow to the treatment plant—an increase in flow has a negative impact on the efficiency of wastewater treatment.

The presence of hormones in surface waters translates into their penetration into groundwater, but also into their accumulation in sediments, with the concentration of these highly exceeding their predicted no-effect concentrations (PNEC), which means the possible risk of harmful effects on aquatic organisms (Rezka et al. 2015).

Biosolids from municipal WWTPs and manure from concentrated animal feeding operations (CAFOs) are commonly used as fertilizer (Raman et al. 2001; Andaluri et al. 2012). However, it should be borne in mind that animal feces are probably the largest source of estrogen hormones in the natural environment (Knight 1980; Tabak 1981; Nachman and Smith 2015; Adeel et al. 2017). Yang et al. (2012) found that biosolids used as agricultural fertilizers can lead to the runoff of steroid hormones, including androgens, estrogens, and progestogens.

The dairy industry has been using growth steroids for years to increase the growth rate of cattle and gain lean muscle mass. Steroid estrogens used in CAFOs have been detected in feces, slurries, and solid waste from cattle, in lagoon effluents, and fertilizers applied directly to agricultural land (Biswas et al. 2013). Poultry, cow, and horse excrements also release significant amounts of steroid estrogens into the environment.

Table 4 lists the concentrations of selected hormones in the environment, including in slurry and swine, dairy farm wastewater, and sow urine. For comparison, observed concentrations in surface waters, groundwater, and drinking water are also given. The concentrations of steroids in agricultural waste and manure are so significant that even small discharges into the environment will increase the concentration of these compounds in water (Aris et al. 2014; Zhang et al. 2014).

**Table 4:** Estrogen concentrations (E1, 17α-E, 17β-E2, and E3) in ng/dm^3^ in the water solutions (based on Adeel et al. 2017)

Sample	Estrone (E1)	17α-estradiol (17α-E2)	17β-estradiol (17β-E2)	Estriol (E3)	Reference
Slurry					
In swine pit	5900–150,000	4000–84,000	1800–49,000	nd	(Li et al. 2010)
In dairy pit	2500–80,000	2000–5000	800–27,000	nd	(Li et al. 2010)
Swine					
Farm effluent	5200–5400	650–680	1000–1500	2200–3000	(Franks 2006)
Manure	70.0	175	15	nd	(Rodriguez-Navas et al. 2013)
Manure leachate	68.1	2.5	nd	nd	(Kjær et al. 2007)
Dairy farm wastewater	370–2356	1750–3270	351–957	nd	(Li et al. 2010)
Lagoon pond	650	nd	nd	nd	(Rodriguez-Navas et al. 2013)
Sow urine	416–490	nd	85–97	127–193	(Zhang et al. 2014)
Grazing land water	78	31	18	nd	(Kolodziej and Sedlak 2007)
1 m deep groundwater	68.1	nd	2.5	nd	(Li et al. 2010)
Surface waters	0.7	nd	nd	nd	(Bolong et al. 2009)
	0.1–3.4	nd	nd	nd	(Włodarczyk-Makuła 2016)
Drinking water	0.4	nd	0.3–2.1	nd	(Bolong et al. 2009)
STP/effluent	12–196	6.4–12.6	6.2–42.2	nd	(Pal et al. 2010)

*nd no data

The study (Schiffer et al. 2001) examined the residue and degradation in solid dung, liquid manure, soil of the steroids trenbolone acetate (TbA) and melengestrol acetate (MGA), compounds approved for use as growth stimulants in livestock. Manure and slurry were spread on corn fields after 4.5 and 5.5 months of storage. In soil samples, trenbolone was detectable up to 8 weeks after fertilization, and MGA was detected even until the end of the cultivation period. It turns out that sex hormones tend to be absorbed in soil and sediments, bioaccumulating (Wee and Aris 2017). Additionally, the EE2 hormone has low volatility and hydrophobic properties, which makes it more resistant to biodegradation—it persists in the environment (Zhang et al. 2014; Wee and Aris 2017; Dai et al. 2022).

Dai et al. (2022) examined the process of sorption and desorption of sex hormones in soil- and sediment–water systems, and the influence of environmental factors on the intensity of these processes. Hydrophobic partitioning interaction, sediment organic matter (SOM), pH, temperature, minerals, and ion strength influence the sorption potential of sex hormones in the soil. Sex hormones can be desorbed and interact with soil particles after manure fertilization. Rainfall characteristics (intensity and duration), irrigation frequency, and tillage methods will have a significant impact on the movement of pollutants in fertilizer-soil–water systems.

Aquaculture is also a potential source of hormones in the aquatic environment (Kolodziej and Sedlak 2007; Sacdal et al. 2020). The most important sex hormones used in fish farming are estrogens and androgens, which can be of natural origin or synthetic (De et al. 2022). The presence of environmental hormones in aquaculture seawater has been documented, with bisphenol A (Lv et al. 2013) or exogenous hormones, such as carp pituitary homogenate, Ovopel, and Ovaprim—essential for successful reproduction (Nica et al. 2022). Estrone, endogenous steroids, testosterone, and androstenedione were detected in sewage from three fish hatcheries at concentrations of approximately 1 ng/dm^3^ (Kolodziej et al. 2004). In the case of fish farming, the transport of pharmaceuticals from the breeding tank to, e.g., groundwater, may take place directly (water from the tank can penetrate the soil) or indirectly through periodic water exchange in breeding ponds (Czech 2012). The occurrence of synthetic and natural estrogenic hormones in aquaculture further underscores the need for continued research of hormone pollution (Rezka et al. 2015).

Kolodziej et al. (2004) showed that sewage from dairy farms, sewage from aquaculture, and surface water with actively spawning fish may contribute to the presence of steroid hormones in surface waters. It has been shown that all these sources can lead to detectable concentrations of hormones in water, while the concentrations of these compounds show high temporal and spatial variability. The endogenous estrogens 17β-estradiol and estrone and the androgens testosterone and androstenedione were present in sewage from dairy farms in concentrations reaching up to 650 ng/dm^3^. Samples from nearby surface waters showed concentrations of approximately 1 ng/dm^3^ of the steroids 17β-estradiol, estrone, testosterone, and medroxyprogesterone.

When discussing the sources of hormones in the aquatic environment, one should be aware that synthetic hormones are also used in agriculture as crop growth enhancers (Adeel et al. 2017). In addition to synthetic hormones, plants also contain naturally occurring non-steroidal compounds—phytoestrogens (isoflavones, lignans, and coumestans), found in wild and cultivated plants. Research has shown that agricultural runoff can contribute to water pollution with hormones. The same will apply to the use of treated wastewater for field irrigation—Pedersen et al. (2005) identified human pharmaceuticals, hormones, and personal care product ingredients in runoff from fields irrigated with treated wastewater. Runoff from agricultural areas is a non-point source polluting water reservoirs—in contrast to sewage discharges from industrial plants and sewage treatment plants, which are point sources (Rearick et al. 2014; Wee and Aris 2017).

Another threat to water resources, especially groundwater, are cemeteries due to the degradation and leaching of organic material (Lautz et al. 2019). Cemeteries can contribute to water pollution through the release of various contaminants. Baum et al. (2022) showed that groundwater is contaminated by necro-leachate, containing ammonia, heavy metals, and other harmful substances. Fiedler et al. confirmed the presence of pharmaceuticals in cemetery drainage, but described the environmental risk as low (Fiedler et al. 2018). The specific impact of hormones from cemeteries on water pollution is not well-documented and further research is needed to understand the extent of this issue.

Landfills are also a source of pollutants, including hormones (Bexfield et al. 2019). Waste classified as hazardous, such as waste from the chemical, agricultural, or pharmaceutical industries, often ends up in landfills. Leachate from landfills may infiltrate into groundwater (Adeel et al. 2017). These objects are recognized as a significant source of environmental estrogens (Gong et al. 2014; Wilk et al. 2019). Illegal waste dumps should also not be forgotten, placed, among others, in forests or wastelands. Failure to protect the substrate leads to rapid transport of hormones deep into the soil. It is also important that the mineralization of contaminants contained in medicines occurs only as a result of waste combustion. Other waste management methods lead only to the transformation or degradation of pollutants (Czech 2012).

The key to assessing the importance of individual sources in the contamination of the aquatic environment with hormones is the development of research and analytical methods enabling the verification of the concentrations of these compounds in water. Raman et al. (2001) suggests the need for acidification and cold storage of environmental samples tested for estrogens and the use of multiple tests for their detection, because in his study no single test met all the desired criteria for speed, sensitivity, and detection of estrogens.

## Influence on health and environment

Without proper water and wastewater purification, drugs/or its metabolites reach soil and water bodies. Due to that, they state danger to biota, to health, and to the environment (Kanama et al. 2018; Barboza et al. 2019; Lima Morais et al. 2019). They affect water quality, ecosystems, water supplies, and human health (Bautista-Toledo et al. 2005). The quantification of pharmaceuticals presence in human blood, plasma, or urine was studied a long time ago (Erny and Cifuentes 2006; Guedes-Alonso et al. 2015). Generally, it can be stated that their potential ecological effects are still little known. But it has been shown that long-term exposure even to low-level concentrations may have toxic effects on terrestrial and aquatic organisms. Pharmaceutical residues may be taken by plants and then transferred to humans or animals via the food chain (Kanama et al. 2018). This material may serve as a food source for water fauna and may influence significant processes like nutrient transformation. In the arable soils, antibiotics may influence plant growth as well as soil microbial activity. Also, the phytotoxicity of pollutants may disturb the phytoremediation process (Carvalho et al. 2014).

Steroids can be characterized as endocrine disruptors. They have a negative influence on the reproductive, nervous, and immune systems of organisms. In the result, they cause an imbalance of the endocrine system. They exhibit toxic effects even at very low concentration (ng/dm^3^) (Kasprzyk-Hordern et al. 2009; Ren et al. 2022). These compounds have the potential to destroy the routine function of the endocrine system, and they also may cause dire health effects (Bozyigit et al. 2022). There is numerous evidence supporting toxic effects of EDCs on living organisms like fish, birds, panthers, and amphibians (Nghiem et al. 2004).

Generally, it can be stated that endocrine-disrupting compounds can influence the function of natural hormones in aquatic and human organisms (Standley et al. 2008). Those compounds affect the synthesis, transport, and metabolism as well as excretion of hormones from the body (Rezka et al. 2015).

In 1980, it was discovered that estrogens influence fish growth. It was the starting point for further, more advanced investigation. Wastewater effluent released into the environment has a negative effect on human health, ecosystem balance, and animals (Behera et al. 2011; Roudbari and Rezakazemi 2018).

Feminization or monoecism phenomena in fish can be caused by estrogen. As a result, reproductive problems may occur. Poisoned fish through the food chain will be passed to higher nexus which finally may increase the incidence of breast and uterine cancer in women and prostate and testicular cancer in men. Besides, sperm count may decrease (Ren et al. 2022).

Even at trace levels, hormones may strongly influence reproduction behaviors (Zeilinger et al. 2009; Runnalls et al. 2013; Dai et al. 2022), gene expressions (Zucchi et al. 2012, 2013), or endogenous hormone levels in fish exposed to this compounds (Runnalls et al. 2013). According to Muz et al. (2012), EDC presence leads to abnormalities in fish as well in some laboratories animals. Drugs in concentration about 0.1 ng/dm^3^ influence fish organisms (Roudbari and Rezakazemi 2018). EDCs are shown to decrease male sperm count, increases in testicular, ovarian, prostate, and breast cancer. As well as they led to reproductive malfunctions (Nghiem et al. 2004). Complex nervous, developmental as well as behavioral abnormalities may occur after exposure to these undesired substances. Generally, new born babies as well as fetuses are more prone to harmful effects of EDCs. Natural estrogenic hormones like estradiol and estrone can be described as the most disrupting endocrine materials (their endocrine-disrupting potential may be even thousand times higher than synthetic materials (like nonylphenol)) (Nghiem et al. 2004).

Even very low estrogen concentrations (below 0.1 ng/dm^3^) influence human reproduction, livestock, and wildlife. It also has a stimulatory effect on breast tumor growth (Roudbari and Rezakazemi 2018). Some research showed that it may influence uterine cancer, ovary cancer, and some other cancer types (Yi et al. 2016).

Natural steroid estrogenic hormones including estrone (E1), 17β-estradiol (E2), estriol (E3) (secreted by humans and animals), and also synthetic 17α-ethinylestradiol (EE2) used by women are classified as having the highest endocrine-disrupting potential. Natural and estrogenic hormones excreted by humans and animals generally have the highest degree of estrogenic activity in water environment (Karapinar et al. 2016).

E2 and EE2 have estrogenic potency (like binding to the human estrogen receptor) (Pauwels et al. 2008a). Estradiol (E2), ethinylestradiol (EE2), and estriol state danger to the environment. EE2 is partially degraded and its remains interact in nature and as a result they become active again. After releasing to water channels, those compounds may harm marine life. It was reported that marine species exposed to endocrine-disrupting compounds showed fertility disorder, rapid femininity in fish, as well as lower weight in Japanese quails. In human male, decreased sperm count was observed and in female bodies the increased change of ovarian and breast cancer was noticed (Schröder et al. 2016; Yasir et al. 2021). WHO established an acceptable daily intake (ADI) limit as 0.05 µg E2/kg body weight. Dosage above 0.3 mg/person/day was linked to changes in some hormone dependent parameters. For E1 and E3, ADI amounted to 0.07 µg/person/day (Caldwell et al. 2010).

Natural estrogenic hormones—estradiol and estrone—recently are the most endocrine-disrupting compounds. In comparison to other synthetic chemicals, its endocrine-disrupting potential may be even thousands times higher. Estradiol in concentration about 1 ng/dm^3^ may result in a distinctive endocrine-disrupting effect in male trout (Lima Morais et al. 2019). The relationship between residual drugs and health problems was demonstrated. Depending on compound type, mental disorders as well as sexual dysfunctions can take place (Lima Morais et al. 2019). In females and males, excess estrogens may lead to birth defects, abnormal sexual development, and problems with nervous and immune systems. They also have cancerogenic properties (Hartmann et al. 2014). Estrone (E1) presence before menopause may result in polycystic ovarian syndrome or cancer (Barboza et al. 2019).

In the European Commission Technical Guidance Document assessed how a preliminary screening-level risk assessment can be established. It can be performed with RQ approach (Ren et al. 2022) (formula 1):

1 $$\mathrm{RQ}=\frac{\mathrm{MEC}}{\mathrm{PNEC}}$$

where:

MEC: measured environmental concentration.
PNEC: predicted no-effect concentration.

The PNEC value can be calculated like below (formula 2):

2 $$\mathrm{PNEC}=\frac{\mathrm{L}(\mathrm{E})\mathrm{C}50\text{ or NOEC} }{\mathrm{AF}}$$

where:

LE50 or LC50: are acute toxicity factors.
NOEC: is the chronic toxicity factor.
AF: comprises the assessment factors.

When L(E)C_50_ data were applied, AF amounted to 1000. When NOEC data were used, the AF value was reaching 100. The RQ factor represents the degree of ecological environmental risk. The higher an RQ value, there is the higher risk of occurring pollutants in the water environment. An RQ value fluctuating between 0.01 and 0.1 indicates low risk, 0.1 to 1 means that there is moderate risk, and value > 1 indicates high risk of presence of the pollutants in the environment. If available toxicity data are sufficient, the PNEC value can be derived using the method of species sensitivity distribution (Ren et al. 2022).

Nowadays, there are plenty of data regarding the estrogen aquatic toxicity. But for steroids, especially androgens, progesterone, and glucocorticoids, only a few PNEW values were established (Ren et al. 2022). For estrone (E1) some localizations RQ value varied between 0.11 and 1.3467, which indicates that E1 may indicate even high risk to aquatic organisms (Ren et al. 2022). Methyltestosterone (MT) can exhibit toxic effects on reproductive development in aquatic organisms and mammals. MT interferes with normal endocrine function and disrupts the structure and function of the microbial community. This compound may also inhibit gonadal development and reduce the reproductive ability of medaka at concentrations higher than 46.8 ng/dm^3^ (Kang et al. 2008). MT metabolism is slow and may be chronic, long term, and cumulative in human organisms. MT residues can affect acne, hirsutism, rough voice, amenorrhea, breast degeneration, changes in sexual desire, and other aspects of virilism in females. Also children’s growth may be affected by the MT presence. It can be stated that MT is highly toxic (PNEC equal to 0.09 ng/dm^3^), so even low content may exhibit a high RQ value (Ren et al. 2022).

Estrogens mainly led to abnormalities in development and reproductive function. Chronic exposure to ethinylestradiol led to the feminization of male fishes through the production of vitellogenin mRNA and protein. Also, it influenced gonadal development and altered oogenesis in fish. The further exposure to estrogens occurring in the water environment led to elevated content of egg yolk precursor, decreased test size, loss of secondary sex characteristics, and intersex (Gonsioroski et al. 2020). The estrogenic effects linked to the concentration of estrogens (estrone, 17β-estradiol, and diethylstilbestrol) were noticed in mosquitofish in China (Huang et al. 2016). Ethinylestradiol influenced metamorphosis and altered sex ratios in frogs within the vulnerable periods of development. Also rats and mice are vulnerable to estrogens present in the environment (Gonsioroski et al. 2020). 17α-ethinylestradiol exposure in mice males led to increased sexual behavior in a dose-dependent manner (Derouiche et al. 2015). Meyer et al. (2019) showed that 17α-ethinylestradiol led to abnormalities in the remodeling of the spinal arteries, increased the placenta weight, and increased the pups large in terms of gestational age. Besides, this compound led to high abortion rates and changed rats’ maternal behavior. Exposure to 17α-ethinylestradiol before puberty led to advancing puberty. Also increased GnRH expression and increased kisspeptin signaling to GnRH neurons were observed (Gonsioroski et al. 2020).

Thyroid hormones—including thyroxine (T4) and 3,3′,5-triiodothyronine (T3)—are present in all vertebrates and have critical role in a wide variety of psychological functions (like embryonic development, cell differentiation, metabolism, and the proliferation of cell) (Yamanaka et al. 2007). Among them, it has been shown that these compounds exhibit toxic effects on fish. T3/T4 addition results in abnormal bone development for larva fish (caudal fin or skull abnormalities) (Zhang et al. 2021).

## Regulations on hormone presence in water bodies

With the growing problem of the presence of hormones in the aquatic environment, it becomes particularly important to introduce monitoring programs, legal regulations, and also promoting public awareness (Hilz and Gore 2023). However, currently all legal regulations are related mainly to the presence of pharmaceuticals, emerging contaminants (ECs) or endocrine-disrupting compounds (EDCs), and not to the hormones themselves. ECs are a large group of chemicals discovered in the environment related with regular human activities (domestic, agricultural, and industrial processes). ECs include over 3000 types of compounds and their derivatives, such as pesticides, fertilizers, heavy metals, microplastics, pharmaceutically active compounds (PhACs), personal care products (PPCPs), and natural and synthetic hormones usually ending up in the environment (Barboza and Gimenez 2015; Puri et al. 2023). It is important to realize that EDCs regulatory policy is challenging due to their diverse effects on the endocrine system and difficulty in establishing cause-and-effect relationships. Multiple stakeholders (industry, public interest groups) with differing perspectives complicate the regulatory process (Hilz and Gore 2023). Under the European Union’s REACH (Registration, Evaluation, Authorisation and Restriction of Chemicals) regulation, companies must provide safety information on chemicals, and the EU has the authority to restrict or ban chemicals with significant risks (Hilz and Gore 2023).

International policy on EDCs is mainly related with the United Nations Environment Programme (UNEP) and the World Health Organization (WHO) under the direction of the International Programme on Chemical Safety (IPCS) (Hilz and Gore 2023). IPCS works on global evaluations of chemical risks and safety concerns, developing consensus papers on risk assessment procedures, and addressing emerging environmental health concerns worldwide. These actions resulted in developing a consensus statement from the European Commission on EDCs which covers their definition, sources of uncertainty, scientific principles for identification, and research needs (Zoeller et al. 2019).

In the USA, estrogens are still not considered in legislation regarding priority substances. But over the years, EDCs gained attention in order to obtain the surface water quality. Since 2009, 17α-ethinylestradiol, 17β-estradiol, estrone, and estriol have been included in the drinking water monitoring programs regarding unregulated compounds (de Albuquerque Pismel et al. 2021). US Environmental Protection Agency (US EPA) has issued a guidance document Best Management Practices for Unused Pharmaceuticals at Health Care Facility that allows to a decrease in the amount of pharmaceutical waste from healthcare and veterinary facilities. The main aim is to decrease the number of pharmaceuticals released to water systems. In the USA, there are events on regional levels like “Great Lakes Earth Day Challenge”—which collected 4.5 million pills for safe disposal. US EPA also gives grants to support the take-back of non-controlled, unused medicines and send them back with adequate laws. A different way to decrease the entry of pharmaceuticals into the environment is establishing best management practices to handle solid wastes and minimize discharge from landfills (World Health Organization 2011). The US EPA supports the development of Managing Pharmaceutical Waste: A 10-Step Blueprint for Health Care Facilities in the USA. In New York, the Drug Management and Disposal Act (issued by the New York State Department of Environmental Conservation) includes information for all pharmacies and retail stores selling drugs to advise consumers on the proper storage and disposal of unwanted medication (World Health Organization 2011). Two other regulatory frameworks related to hormones monitoring in water can be distinguished: Title 22 Regulations, CA Water Boards Recycled Water Policy and Unregulated Contaminant Monitoring Rule (UCM3 from US EPA) (Hilz and Gore 2023).

In Canada, there were established stewardship programs for household pharmaceutical wastes (at the provincial level) or in some cities which have options to return pharmaceuticals to community pharmacies for safe disposal (World Health Organization 2011).

According to Australian National Guidelines for Water Recycling, a reference value of 17α-ethinylestradiol was assessed as 1.5 ng/dm^3^. This is much lower than one established for 17β-estradiol, estrone, and estriol which are equal to 175 ng/dm^3^, 30 ng/dm^3^, and 50 ng/dm^3^, respectively. In Australia, the regulation of estrogens still not guarantees the high quality of its surface waters. The above concentrations may support the regulation of these compounds in Australian surface and drinking waters (but also in other countries which have not established concentration limits) (de Albuquerque Pismel et al. 2021). The main goal of the RUM Project implemented in Australia is to inform consumers about appropriate options for drug disposal. Also, the Environmental Protection Authority and the National Health and Medical Research Council established guidelines on the management of waste generated in health-care facilities. It was also stated that if it is possible, pharmaceutical waste should be incinerated and not sent to landfills or released to sewers (World Health Organization 2011).

The Brazilian Association of Sanitary and Environmental Engineering—São Paulo Section (ABES/SP 2012) published a document including priority substances. Only 72 substances were included in this document—estrogens were selected during the first stage of selection but they were not included in the final list because the current knowledge of its toxicity does not allow to establish reference doses that can be used to establish water quality criteria (de Albuquerque Pismel et al. 2021).

In Europe, there are various standardized take back programs regarding pharmaceuticals. In the current days, the European legislation draws attention to the decreasing the micropollutant content in the environment (among others ETAP—Environmental Technologies Action Plan highlights the importance of water quality). European Union included estradiols: 17β-estradiol (E2) and 17α-ethinylestradiol (EE2) and diclofenac on the watch list (Directive 2013/39/EU). For diclofenac, standard values of 100 ng/dm^3^ for inland waters and 10 ng/dm^3^ for coastal water were proposed (Schröder et al. 2016). E2, EE2, and estrone were included in the watch list published in Executive Decision 2015/495 (Le Coadou et al. 2017). In 2018, this decision was repealed and replaced by a new watch list—Executive Decision 2018/840. E2 and EE2 have been maintained in this document but they were not taken into account in surface water quality monitoring legislation (de Albuquerque Pismel et al. 2021). COM (2017)753 document proposed the including three EDCs in 2020/2184 Directive; the substances are 17β-estradiol, nonylphenol, and bisphenol A. But probably only bisphenol A will be included. 17β-estradiol and nonylphenol are expected to be added to the European Union watch list in case when more monitoring data will be obtained. In the EU, some countries have already given guidelines that consider the maximum permissible level of above-mentioned substances in drinking water (de Albuquerque Pismel et al. 2021). One of the way to control pollutants appearing in water is the European Union’s REACH (Registration, Evaluation, Authorisation and Restriction of Chemicals) regulation, according to which companies must provide safety information on chemicals, and the EU has the authority to restrict or ban chemicals with significant risks (Hilz and Gore 2023).

## Conclusions

The literature review on the occurrence and sources of hormones in water resources allowed drawing the following conclusions:

- Estrone, estradiol, estriol, and ethinylestradiol are the most common natural and synthetic estrogens.
- Estrogens in the environment can be found in the environment in concentrations from 0.1 ng/dm^3^ (surface water) even up to 150,000 ng/dm^3^ in slurry.
- Steroid hormones (e.g., estrogens, androgens, progestogens, glucocorticoids, and mineralocorticoids) are frequently found in the environment, and water contamination by those compounds has become a worldwide problem.
- Standard wastewater treatment technologies are not sufficient in removing hormones from treated wastewater and implementation of more advanced techniques is needed.
- Although hormones are released into the environment naturally with living organisms’ excretion, significant amounts of these substances come from anthropogenic sources. Most of them are associated with municipal, industrial, hospital sewage, and wastewater treatment plants. However, other significant sources of hormone pollution include animal breeding, agriculture and fish farms (aquaculture), veterinary offices, medical universities, and care facilities, or irresponsible medical flushing. Also pharmaceutical and cosmetic industry, as well as production processes, may lead to hormone release to the water bodies. In the literature, the rarest mentioned sources of hormones are cemeteries and landfills.
- Presence of hormones in the environment is related to various negative effects, like lower plant material biodegradation, influence on nutrient transformation, and influence on plant growth as well as soil microbial activity. Hormone presence also impacts birth defects, abnormal sexual development, and problems with nervous and immune systems. They also have cancerogenic properties. Estradiol and estrone are stated to have the most endocrine-disrupting properties.
- The presence of hormones in the aquatic environment requires introducing monitoring programs, providing legal regulations, and improving public awareness. The main issue with current legal regulations is that they consider generally the presence of pharmaceuticals and not particularly the hormones. Nowadays, there are no legal acts related only to hormone limitation and monitoring in the aquatic environment. International policy on endocrine-disrupting compounds is mainly related with the United Nations Environment Programme (UNEP) and the World Health Organization (WHO) under the direction of the International Programme on Chemical Safety (IPCS). However, there are also regulations in specific countries (e.g., in USA, Brazil, Australia, and European countries).

## Data Availability

Data sharing is not applicable to this article as no new data were created or analyzed in this study.
